# Use of extended curettage with osteotomy and fenestration followed by reconstruction with conservation of muscle insertion in the treatment of Enneking stage II locally aggressive bone tumor of the proximal extremities: resection and treatment of bone tumors

**DOI:** 10.1186/1477-7819-11-54

**Published:** 2013-03-05

**Authors:** Feiyan Chen, Jun Xia, Siqun Wang, Yibing Wei, Jianguo Wu, Gangyong Huang, Jie Chen, Jingsheng Shi

**Affiliations:** 1 Department of Orthopedics, Huashan Hospital, Fudan University, Shanghai, 200040, China

**Keywords:** Bone tumor, Conservation of muscle insertion, Extended curettage, Proximal extremity, Reconstruction, Resection

## Abstract

**Background:**

The purpose of this study was to investigate the clinical efficacy of extended resection with osteotomy, fenestration and conservation of muscle (tendon) insertion in the treatment of bone tumors.

**Methods:**

A total of 15 patients with locally aggressive bone tumors (Enneking stage II) in the adjacent muscle (tendon) insertion of the proximal extremity were enrolled in the present study (mean age of 29 years). Extended curettage of lesions with osteotomy, fenestration and/or conservation of muscle (tendon) insertion and internal fixation with a bone graft or bone cement was performed at stage I. Postsurgical brace protection was used for 4 to 12 weeks and the patients were periodically followed-up by X-ray and functional assessment. Recurrence, postsurgical Enneking score and outcome rating were assessed.

**Results:**

Treated cases included 15 patients aged 29 ±7.75 years (range, 18 to 42) with a male to female ratio of 8:7. Six had a femoral tumor and nine had a humeral tumor. These tumors comprised three chondroblastomas, five giant-cell tumors and seven aneurysmal bone cysts. Follow-up for 48 ±12.95 months (range, 25 to 72) revealed that 13 of 15 (87%) patients exhibited no recurrence. Local recurrence was observed in a patient with an aneurysmal bone cyst (nine months) and one with a giant-cell tumor (12 months). Mean Enneking scores were 27 ±4.07 (range, 18 to 29). Except for the patient with the recurrent giant-cell tumor, all patients reported good (13%, 2 out of 15) or very good (80%, 12 out of 15) outcomes. Very good outcomes were reported in 92% of patients (12 out of 13) without recurrence.

**Conclusions:**

The procedures used in this study achieved high clinical efficacy, complete lesion removal, reduced recurrence and good restoration of joint function in patients with primary locally aggressive Enneking stage II bone tumors of the proximal extremities.

## Background

Locally aggressive bone tumors are a group of commonly recurrent and metastatic bone tumors that predominantly occur in the epiphysis of the long bone of the adjacent joints, including giant-cell tumor (GCT) of bone, aneurysmal bone cyst (ABC), chondroblastoma (CBT) and osteoblastoma [1]. According to Enneking surgical staging, progression of these tumors can be understood in terms of tumor stage [2]. Using this scale, stage I represents the latent phase. At stage II, tumors become active, exhibiting expansive growth and thinning of the bone cortex within the compartment. Stage III is the most aggressive, with lesions piercing through the bone cortex and involving soft tissues surrounding the compartment. Routine treatment includes surgical therapy with extended curettage (IC) of lesions, local adjuvant treatment, graft implantation and effective internal fixation [1]. Because most lesions are adjacent to the joint and muscle (tendon) insertion, IC of lesions may damage muscle (tendon) insertions, exerting detrimental effects on postsurgical limb function. If protection is limited to the muscle (tendon) insertion, a sufficient operative field is difficult to obtain, preventing effective curettage. Therefore, improved methods for treatment selection are required for more effective and successful treatment of locally aggressive musculoskeletal tumors. The current status of treatment and recurrence of these tumors, as discussed in the present study, are briefly reviewed.

### Treatment of aneurysmal bone cysts

ABCs are relatively rare in primary bone tumors, constituting only 1% to 2% of all primary bone tumors. These tumors exhibit rapid growth and high invasiveness, and are often destructive to surrounding tissues [3]. Ubiquitin-specific protease 6 has been implicated in the development of ABCs [3,4], and treatment with curettage and bone grafting or bone cementation has been reported to achieve 70% to 90% success with 10% to 30% recurrence rates [3,5]. IC, or aggressive curettage, applies drills, local adjuvant therapies (such as cauterization), phenolic therapies or cryotherapies to reduce the rate of local recurrence [3,5,6]. ABCs of specific sites, such as the vertebral body and pelvis, can be treated with sclerosing therapies using percutaneous injections of Ethibloc, ethanol and methylprednisolone. In addition, selective arterial embolization has been recommended as an alternative therapy [7].

### Treatment of chondroblastomas

A CBT is a rare cartilage-derived bone tumor that constitutes 1% of all benign bone tumors. It predominantly affects young men and often exhibits invasiveness or malignant behavior [8,9]. Following recommended surgical treatments, the two- to three-year recurrence rate is as high as 10% to 20%, which may partially be due to wide application of inappropriate surgical methods [9,10]. IC, however, has been demonstrated to reduce local recurrence rates [9-11]. IC has been employed for the treatment of bone CBT, producing a recurrence rate of only 11% (2 out of 18) in one study [11]. In another study of 25 patients with a CBT, IC produced a recurrence rate of only 4.2% (1 out of 24) over an eight-year follow-up period [8].

### Treatment of giant-cell tumors of bone

GCTs of bone constitute 4% to 8% of all primary bone tumors and predominantly occur in 20- to 40-year-old (middle-aged) individuals [12]. Recently, biotherapy with receptor activator of nuclear factor kappa-B ligand monoclonal antibodies and colony-stimulating factor 1 signaling pathway inhibitor has been introduced for clinical treatment of GCT [13], though optimal clinical treatment still depends on surgery. Conventional intralesional curettage has been reported to result in recurrence rates higher than 30% in GCT [12,14], whereas wide excision or segmental tumor resection has been reported to produce much lower recurrence rates of only 7% to 10%. These methods, however, require complex bone and soft tissue repair and reconstruction combined with revision surgery following segmental resection of tumors. Thus, the high rate of complications associated with these procedures necessitates careful consideration of the risk of recurrence versus maximum conservation of joint function [14,15].

Both primary and recurrent GCT of bone that are classified as Enneking stage II are conventionally treated with IC, and joint function surrounding the lesion is generally conserved to the highest possible extent [2,13,16]. In addition, IC has been widely recommended for the treatment of cyst walls [2,12,16,17]. A retrospective analysis of 349 GCT cases demonstrated that IC significantly reduced the rate of local recurrence of Enneking stage II and partial stage III tumors to only 11.1% [17].

Although extended resection with conservation of the bone cortex has been previously applied to treat locally aggressive bone tumors in the extremities, the treatment strategy proposed in this study mainly aimed to address the problem of bone cortex reservation and conservation of muscle (tendon) insertion. The clinical efficacy of extended resection with osteotomy, fenestration and conservation of muscle (tendon) insertion for treatment of musculoskeletal tumors was investigated in patients with locally aggressive musculoskeletal tumors including ABC, CBT and GCT types. Rates recurrence and functional restoration was assessed, providing potential prognostic indicators that may aid in the clinical selection of treatments for patients with locally aggressive musculoskeletal tumors.

## Methods

### Participants

From 2004 through 2009, a total of 29 patients with locally aggressive bone tumors of the extremities were admitted to our hospital. Of these, only those exhibiting tumors in the adjacent muscle (tendon) insertion of the proximal extremities humerus or femur were included in the present study. All included patients also exhibited an Enneking stage of II. Patients were excluded if they presented signs of malignant bone tumor according to presurgical pathological examinations using fine needle aspiration cytology or open biopsy.

For each included patient, bone cortex injury at the lesion site was identified according to presurgical X-rays and computed tomography (CT) scans, and the extent of each lesion was identified using magnetic resonance imaging (MRI).

### Surgical procedures

For all patients, brachial plexus anesthesia, lumbar anesthesia or general anesthesia was administered according to the lesion site, and intravenous antibiotics were administered 30 minutes prior to the surgery.

Each patient exhibiting a tumor of the proximal femur was placed in the lateral position, and the insertion of the abductor muscles (gluteus medius and minimus muscles) and vastus lateralis muscle were exposed using the lateral femoral approach (resection of biopsy tissues). The insertion of the abductor muscle was maintained intact, and osteotomy was performed via the greater trochanter. The greater trochanter bone flap was uncovered to expose lesions. Lesion tissues in the intertrochanteric area and femoral neck were completely excised by scraping with a curet at various angles until the bursa wall was reached, and the grooved region of the bony crest was progressively removed with a high-speed drill. The bursa wall is the inner wall or bony crest of the lesion, representing the boundary of aggressive bone tumors. Extended curettage requires thorough curettage of the lesion tissue including the residual lesions in the groove of the bursa to achieve complete excision of the lesion.

**Figure 1:**
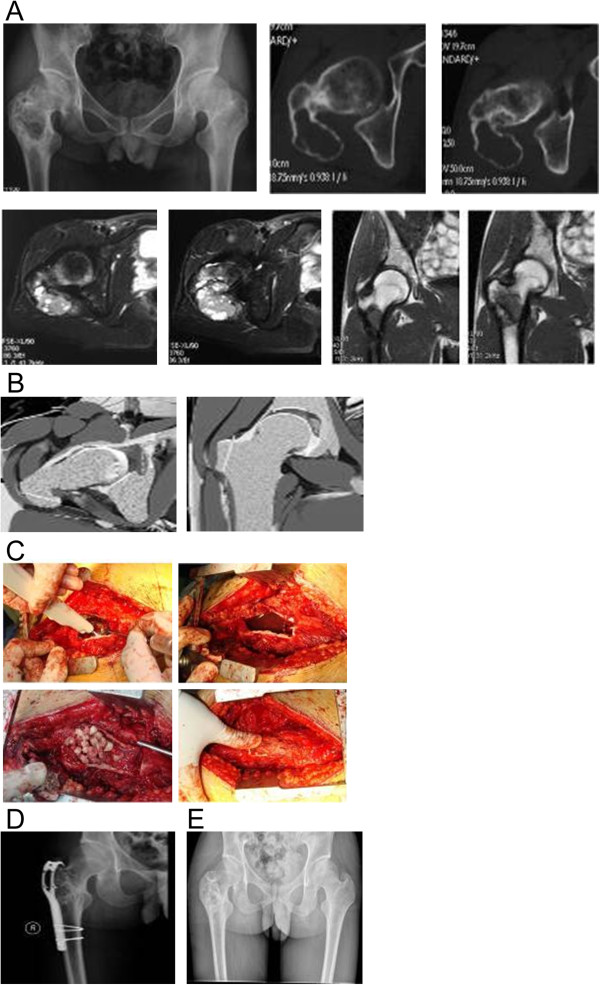
**Male patient, 19 years old with an aneurysmal bone cyst of the right proximal femur. (A)** Presurgical X-ray and computed tomography scan showing expansive cystic change in lesions in the right proximal femur, characterized by bony partition and thinning bone cortex. Presurgical magnetic resonance imaging indicates that lesions exhibit unequal signal intensities with lobular changes. **(B)** Sketch map of presurgical osteotomy line with osteotomy line (red) showing the greater trochanteric osteotomy and conserved insertion of the gluteus medius and minimus muscles. **(C)** Intraoperative cauterization using an argon gas knife, anhydrous ethanol immersion, mixed bone graft with autogenous bone, and artificial bone and reduction osteotomy. **(D)** X-ray at postsurgical month 24 revealing scattered calcifications on the graft regions in the proximal femur without right hip pain. **(E)** Internal fixation removed after postsurgical month 36. X-ray revealing scattered calcifications on the graft regions of the right hip, not significantly different from 24-month X-rays. No hip pain, normal weight-bearing, good Enneking score and normal hip joint activity were reported.

The wound was cauterized using an argon gas knife followed by normal saline washing. This procedure was repeated three times. Femoral head and neck areas beyond the reach of drill and knife techniques were further treated by immersion in anhydrous ethanol for 15 min. Greater trochanter bone flap lesions on the medial side and bony surface were treated similarly (Figure 1B,C).

**Figure 2:**
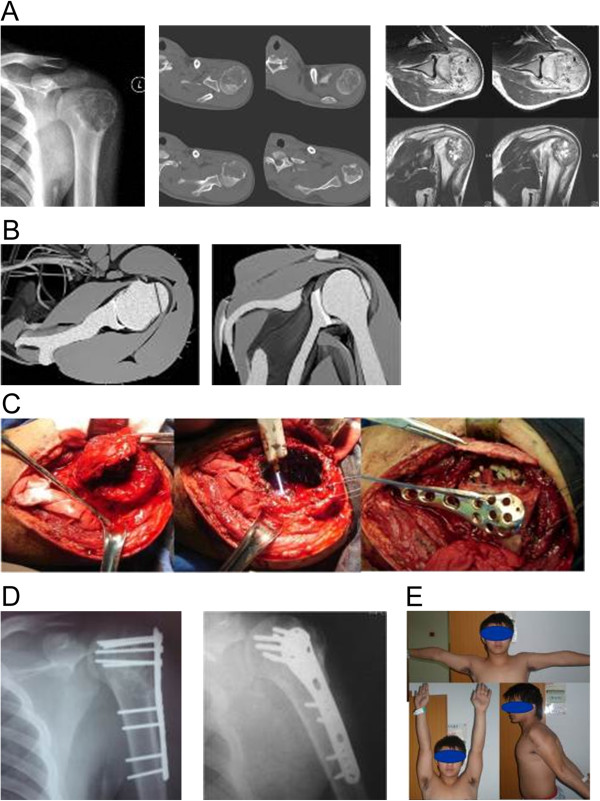
**Male patient, 19 years old, with chondroblastomas of the left proximal humerus. (A)** Presurgical X-ray and computed tomography scan showing expansive lesion growth in the proximal humerus and thinning of the bone cortex with bony septum and scattered calcifications. Presurgical magnetic resonance imaging indicates T2-weighted images of tumors with inhomogeneous moderate signals and scattered high signals complicated by edema and swelling of the surrounding soft tissues and the rotator cuff insertion adjacent to tumors. **(B)** Sketch map of osteotomy of the greater tuberosity of the humerus showing osteotomy line (red) without injury to the subscapularis muscle, infraspinous muscle, or insertion of the supraspinatus muscle. **(C)** Intraoperative treatment showing osteotomy and fenestration performed via the greater tuberosity of the humerus and repeated cauterization with an argon gas knife. Autogenous and artificial bones were grafted. The defective region of the aneurysm shell was then covered with autologous iliac bone containing cortical bone and treated with internal fixation using an anatomical titanium alloy plate. **(D)** X-ray at postsurgical months 12 and 24 revealing no local recurrence. **(E)** Internal fixation was removed after 24 months, resulting in normal left shoulder joint function, good Enneking score and no pain.

Each patient exhibiting a tumor of the proximal humerus involving the intertubercular regions was placed in the beach chair position, and an incision was made on the anterior third of the deltoid muscle prior to resection of biopsy tissues. The rotator cuff insertion was maintained intact, and osteotomy and fenestration of the greater trochanter were performed. The muscle bone flap was uncovered and lesions were excised using the same method previously described for a tumor of the proximal femur (Figure 2B,C).

After complete lesion curettage of tumors of either the proximal femur or humerus, autogenous iliac cancellous bone grafts and/or artificial bones were implanted immediately after tumor resection in the same surgery (stage I), rather than performing functional reconstruction in a separate operation. Bone cement augmentation was routinely administered according to standard protocols, particularly for patients with GCT. The proximal femur was fixed with a titanium alloy plate. The proximal humerus was fixed with anatomical humeral plates and screws (Figures 1C and 2C).

### Postsurgical treatment

All patients were administered continuous intravenous antibiotics for 24 h after surgery, and drainage tubes were removed between 24 h and 48 h after surgery. Postsurgical bracing was applied for four to six weeks after surgery. Isometric muscle strength training of the upper extremity was performed within 24 h after surgery in most patients.

### Follow-up

All patients underwent follow-up after surgery for a minimum of two years, and the time to full weight-bearing was recorded for each patient. Periodic postsurgical X-rays were performed to monitor bone healing and track local recurrence. Imaging investigations, including MRI, were used to confirm the recurrence of lesions. During the follow-up period, CT and MRI were performed only if deemed necessary due to abnormal X-ray or functional assessments. Musculoskeletal tumors were evaluated using the Enneking postoperative Musculoskeletal Tumor Society score consisting of 30 total possible points [1]. More than 70% of functional recovery was rated very good, 60% to 70% of recovery was rated good, 50% to 60% of recovery was rated moderate and less than 50% of recovery and amputation or death was defined as poor.

### Statistical analysis

All data were analyzed using SPSS version 11.0 (SPSS Inc., Chicago, IL, USA). Quantitative data for age, follow-up duration and Enneking score data are expressed as means ±SD.

## Results

### Demographic and clinical characteristics of included patients

Of 29 patients treated, 15 presented with locally aggressive tumors of the proximal femur (six patients) or humerus (nine patients), including eight men and seven women with a mean age of 29 ±7.75 years (range, 18 to 42). An ABC was diagnosed in seven patients including five primary and two recurrent patients; CBT was diagnosed in three patients; and GCT of bone was observed in five patients. Tumors in the proximal humerus were observed in six patients, and tumors in the proximal femur were found in nine patients (Table 1).

**Table 1:** Characteristics and follow-up outcomes of 15 malignant musculoskeletal tumor patients

	**Gender**	**Age (years)**	**Site**	**Diagnosis**	**Treatment**	**Follow-up (months)**	**Recurrence (months after surgery)**	**Enneking score**	**Rating of score**
	F	22	PH	ABC	IC+BG	37	-	29	Very good
	M	19	PF	ABC	IC+BG	47	-	29	Very good
	M	22	PH	ABC	IC+BG	36	-	29	Very good
	F	27	PF	ABC-R	IC+BG	72	9	20	Good
	M	21	PF	ABC	IC+BG	35	-	28	Very good
	F	28	PF	ABC-R	IC+BG	45	-	29	Very good
	M	27	PF	CBT	IC+BG	52	-	29	Very good
	M	37	PF	GCT	IC+BC	46	-	29	Very good
	M	19	PH	CBT	IC+BG	54	-	29	Very good
	F	33	PF	GCT	IC+BC	43	-	28	Very good
	F	38	PF	GCT	IC+BC	66	12	18^a^	Moderate^a^
	F	42	PH	GCT	IC+BC	53	-	29	Very good
	M	38	PH	CBT	IC+BG	45	-	28	Very good
	F	26	PH	ABC	IC+BG	25	-	19	Good
	M	36	PF	GCT	IC+BC	67	-	29	Very good
**Totals**	M 8/15 (53%)	29 ±7.75 (18 to 42)^b^	PF: 6/15 (40%)	ABC: 7/15 (47%)^c^	IC+BC: 5/15 (33%)	48 ±12.95 (25 to 72)^b^	Two	27 ±4.07 (18 to 29)^b^	Very Good 12/15 (80%)
	F 7/15 (47%)		PH: 9/15 (60%)	CBT: 3/15 (20%)	IC+BG: 10/15 (66%)	PH: 42 ±11.2 (25 to 67)^b^		PH: 27 ±4.3 (19 to 29)^b^	
				GCT: 5/15 (33%)		PF:53 ±12.7 (35 to 72)^b^		PF: 27 ±4.0 (18 to 29)^b^	

ABC, aneurysmal bone cyst; ABC-R, recurrent aneurysmal bone cyst; BC, bone cement augmentation; BG, bone grafting; CBT, chondroblastoma; F, female; GCT, giant-cell tumor of bone; IC, extended curettage; M, male; PH, proximal humerus; PF, proximal femur.

^a^Postsurgical scores and ratings after recurrence prior to secondary surgery; ^b^mean ±SD (range); ^c^two recurrent cases.

### Proximal tumor and bone flap characteristics

In proximal femur tumors, most lesions were widely distributed in the intertrochanteric areas and femur neck, whereas proximal humerus tumors demonstrated expansive growth in the humeral head, leading to complications related to thinning bone cortex (Figures 1A and 2A). No lesions penetrated the bone cortex, and the surface of the articular cartilage remained intact in all patients. Soft tissue edema without lesion infiltration was observed in some cases. The bone flap with tendon insertion was reduced in all cases, and small bone defects were present in three patients following lesion curettage.

After the tumor-shell bone was treated at the osteotomy site, remaining small bone defects were covered with sheets of autogenous iliac bone containing the cortical bone. For the cases with GCT of bone, the cavity was filled with bone cement, whereas the cavity of other cases were filled with autogenous cancellous bone and/or artificial bone grafts.

### Postsurgical outcomes

The mean follow-up for all patients was 48 ±12.95 months (range, 25 to 72). During this period, no short-term complications, such as hematoma or infection, were observed. Furthermore, no long-term complications were observed, including pathological fractures, local malignant transformation or distant metastasis. Hip and joint activity gradually improved in patients treated with bracing during the two weeks following surgery, and all braces were removed between four and six weeks after surgery. Most patients were able to initiate isometric muscle strength training of the lower extremity within 24 h of surgery. Patients with femoral tumors began partial weight-bearing and walking with the aid of crutches at eight weeks, and all braces were removed by postsurgical week 12.

One case of recurrent ABC was treated with curettage of lesions with fenestration underneath the tuberosity prior to IC with fenestration, resulting in hip pain within six months of surgery. X-ray follow-up at postsurgical month 12 revealed local bone graft absorption and slight hip pain in one patient, particularly when walking, that persisted at postsurgical month 36. A second surgery was advised. The patient thought that the hip pain was tolerable and could normally walk with weight-bearing. They did not undergo surgery and is currently being followed-up.

### Tumor recurrence

Local recurrence was observed in only two of fifteen patients (13%), including at nine months in one patient with ABC of the proximal femur, who underwent a second surgery and at 12 months in one patient with GCT. The patient with recurring GCT underwent secondary IC and bone cement augmentation. In the postsurgical follow-up of the secondary surgeries, no additional recurrence was reported. No local recurrence was detected in any of the other 13 patients (Table 1).

### Enneking scores

**Figure 3:**
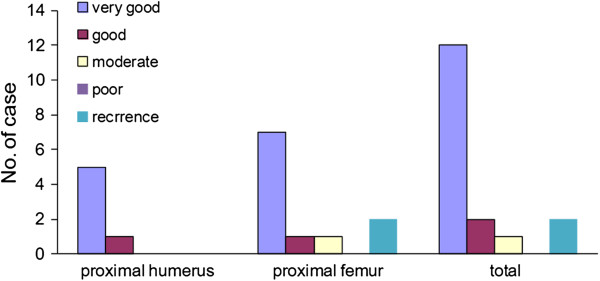
**Postsurgical follow-up of Enneking score and recurrence. ***Enneking score ratings*: Very good (>70); good (60 to 70); moderate (50 to 60); and poor (<50).

The mean Enneking score for all patients was 27 ±4.07 (18 to 29). The mean Enneking score for upper limbs was 27 ±4.0 (18 to 29) and lower limbs was 27 ±4.3 (19 to 29). The lowest Enneking score was reported in the patient with recurrent GCT. Except for this patient, all patients reported good or very good outcomes. Very good outcomes were reported in 80% of all patients (12 out of 15) and 92% of patients (12 out of 13) without recurrence (Table 1 and Figure 3).

## Discussion

Patients with locally aggressive bone tumors such as ABC, GCT and CBT were effectively treated with IC (aggressive curettage) and high-speed drilling in combination with local adjuvant treatment measures, including repeated cauterization of the cyst wall with an argon gas knife and anhydrous alcohol immersion of deep regions. These findings indicate that successful treatment of locally aggressive bone tumors of Enneking stage II is possible using these methods in practical clinical settings. In the current study, the postsurgical recurrence rate was 13.3% (two out of fifteen), much lower than the postsurgical recurrence reported by several similar studies [3,11,12,14]. Therefore, IC may also effectively reduce the postsurgical local recurrence of bone tumors of each of these types.

Following IC treatment, satisfactory functional restoration was achieved by all patients in the current study. Using IC to treat CBT of bone, van der Geest *et al*. [11] reported a postoperative Musculoskeletal Tumor Society score of 93% (28 out of 30). In another study, IC for treatment of 24 cases of CBT resulted in 87.5% very good or good functional outcomes according to Enneking scores (28 to 30) [ 8]. These treatments, however, did not consider the importance of the muscle (tendon) insertion. The current method allows for optimal conservation of the bone context and avoids intraoperative damage to the muscle (tendon) insertion. In the proximal femur, for example, when the muscle insertion is protected during surgery, traditional curettage can be performed with fenestration underneath the tuberosity or on the posterior femoral neck. The relatively small operating field and complexity of the operation can result in incomplete resectioning of tumors that widely involve the intertrochanteric area and femoral neck. As a result, the risk of postsurgical recurrence and osteonecrosis of the femoral head is high.

The current study employed the surgical techniques described by Ganz *et al*. [18], who suggested that lesion exposure could be effectively achieved through greater tuberosity osteotomy. This strategy not only protects the muscle insertion but also provides a larger bone window (about 5 cm × 3 cm) that increases the operating field view. This strategy is optimal for achieving IC of lesions in the intertrochanteric areas and femoral head (Figure 2B,C). In the present study, imaging studies confirmed that the anatomical structures of the rotator cuff insertion, curettage of lesions with osteotomy and fenestration of the greater tubercle of humerus achieved good exposure and conserved the rotator cuff insertion, particularly in tumors involving the proximal humerus (Figure 1B,C).

After complete treatment of the lesions using the technique applied in the current study, bone fragments on the muscle insertion were reduced by applying effective internal fixation. This IC method was designed to conserve the muscle insertion integrity, thus enabling reconstruction of bone-bone fixation but not tendon-bone fixation following curettage. As a result, postsurgical healing was primarily bone healing instead of tendon-bone healing, which may produce more pronounced functional impairments.

In the present study, postsurgical follow-up revealed that about 93% of patients achieved very good or good functional results according to Enneking scores, which is similar or superior to the results of previous studies [8,11,19]. Although these results indicate that the current surgical strategy is superior for conservation of joint function and postsurgical rehabilitation in patients with bone tumors of several types, it is important to consider that this study also has some limitations. In particular, the small number of patients and the use of only a single-center patient population, which may not be representative of general patient populations due to the specialized nature of the treatment facility, must be considered. Furthermore, early functional exercise following surgery and brace protection may have also impacted these positive findings. These techniques, however, merit further study in larger cohorts and more varied patient populations due to their promising potential for dramatically reducing recurrence rates and enhancing functional outcomes. In addition, application of this technique for secondary surgery for recurrent tumors should be more carefully explored, though positive results can be expected based on these preliminary findings.

## Conclusions

Effective treatment of locally aggressive bone tumors requires comprehensive analysis of the characteristics, site and aggressiveness (Enneking stage) of tumors. When determining an appropriate treatment strategy, clinicians should carefully weigh the benefits of function conservation in the proximal limb and joint with complete tumor resection. The results of previous studies and the currently reported study suggest that extended resection of tumors with osteotomy, fenestration and conservation of muscle (tendon) insertion can be combined with effective internal fixation to reduce postsurgical recurrence and protect joint function. Thus, this strategy may be effective and feasible for treating primary or recurrent locally aggressive bone tumors classified as Enneking stage II in the proximal extremities, particularly those of the femur and humerus.

## Consent

Written informed consent was obtained from the patients for publication of this report and any accompanying images.

## Abbreviations

ABC: aneurysmal bone cyst; CBT: chondroblastoma; CT: computed tomography; GCT: giant-cell tumor; IC: extended curettage; MRI: magnetic resonance imaging.

## Competing interests

The authors declare that they have no competing interests.

## Authors’ contributions

Xia Jun and FeiYan Chen designed the study protocol, Xia Jun, YiBing Wei, SiQun Wang and FeiYan Chen performed the operations. SiQun Wang, JianGu Wu, and GangYong Huang participated the postoperative care, follow-up work and data statistics. Jie Chen and JingSheng Shi performed the literature research and manuscript preparation. All authors read and approved the final manuscript.
